# Beyond cyanotoxins: increased *Legionella*, antibiotic resistance genes in western Lake Erie water and disinfection-byproducts in their finished water

**DOI:** 10.3389/fmicb.2023.1233327

**Published:** 2023-08-28

**Authors:** Jiyoung Lee, Seungjun Lee, Chenlin Hu, Jason W. Marion

**Affiliations:** ^1^Division of Environmental Health Sciences, College of Public Health, The Ohio State University, Columbus, OH, United States; ^2^Department of Food Science and Technology, The Ohio State University, Columbus, OH, United States; ^3^Infectious Diseases Institute, The Ohio State University, Columbus, OH, United States; ^4^Department of Food Science and Nutrition, Pukyong National University, Busan, Republic of Korea; ^5^College of Pharmacy, University of Houston, Houston, TX, United States; ^6^Department of Public Health and Clinical Sciences, Eastern Kentucky University, Richmond, KY, United States

**Keywords:** trihalomethane, Lake Erie, haloacetic acids, microcystin, *Microcystis*, mobile genetic elements, antibiotic resistance, *Legionella*

## Abstract

**Background:**

Western Lake Erie is suffering from harmful cyanobacterial blooms, primarily toxic *Microcystis* spp., affecting the ecosystem, water safety, and the regional economy. Continued bloom occurrence has raised concerns about public health implications. However, there has been no investigation regarding the potential increase of *Legionella* and antibiotic resistance genes in source water, and disinfection byproducts in municipal treated drinking water caused by these bloom events.

**Methods:**

Over 2 years, source water (total *n* = 118) and finished water (total *n* = 118) samples were collected from drinking water plants situated in western Lake Erie (bloom site) and central Lake Erie (control site). Bloom-related parameters were determined, such as microcystin (MC), toxic *Microcystis*, total organic carbon, N, and P. Disinfection byproducts (DBPs) [total trihalomethanes (THMs) and haloacetic acids (HAAs)] were assessed in finished water. Genetic markers for *Legionella*, antibiotic resistance genes, and mobile genetic elements were quantified in source and finished waters.

**Results:**

Significantly higher levels of MC-producing *Microcystis* were observed in the western Lake Erie site compared to the control site. Analysis of DBPs revealed significantly elevated THMs concentrations at the bloom site, while HAAs concentrations remained similar between the two sites. *Legionella* spp. levels were significantly higher in the bloom site, showing a significant relationship with total cyanobacteria. Abundance of ARGs (*tet*Q and *sul*1) and mobile genetic elements (MGEs) were also significantly higher at the bloom site.

**Discussion:**

Although overall abundance decreased in finished water, relative abundance of ARGs and MGE among total bacteria increased after treatment, particularly at the bloom site. The findings underscore the need for ongoing efforts to mitigate bloom frequency and intensity in the lake. Moreover, optimizing water treatment processes during bloom episodes is crucial to maintain water quality. The associations observed between bloom conditions, ARGs, and *Legionella*, necessitate future investigations into the potential enhancement of antibiotic-resistant bacteria and *Legionella* spp. due to blooms, both in lake environments and drinking water distribution systems.

## Introduction

Changes in the global climate have been linked to increasing eutrophication and an increase in frequency, duration and intensity of harmful algae blooms (Glibert, 2020). Among these blooms, freshwater blooms are dominated by cyanobacterial blooms, in which most are associated with the production of cyanotoxins (Loftin et al., 2016). These blooms, pose health risks by compromising drinking water sources, food production, aquatic ecosystems, and recreational waters (Brooks et al., 2016; Lee J. et al., 2017; Mrdjen et al., 2018). Microcystins (MCs), prevalent cyanotoxins, are commonly detected in freshwater used for drinking water supplies, irrigation, recreation, and fish farms (Loftin et al., 2016; Lee S. et al., 2017; Mrdjen et al., 2018). Epidemiological and animal studies have linked exposures to bloom-affected water to an increased frequency of non-alcoholic liver diseases, liver cancer, neurodegenerative diseases, gut microbiome disturbance, and more progressed liver cancer (Zhang et al., 2015; Lee et al., 2019, 2020, 2022; Gorham et al., 2020).

Lake Erie in the United States has witnessed recurring severe cyanobacteria blooms, particularly the massive 2011 event spanning 5,000 km^2^ (Stumpf et al., 2016; Zhang et al., 2017; Jankowiak et al., 2019). Elevated MCs exceeding 1 μg/L in finished drinking water in 2013–2014 prompted “Do Not Drink” advisories (Stumpf et al., 2016). Effective drinking water treatment is pivotal for safeguarding public health from cyanotoxins, necessitating robust strategies for MCs and cyanotoxin removal in bloom-impacted source water beyond source protection measures. While MCs are effectively removed using disinfection with free chlorine or ozone followed by additional activated carbon treatment (Cheung et al., 2013; Gonsior et al., 2019) in most cases, little is known about disinfection by-products (DBPs) that can form via chlorination in the Lake Erie region from MCs and other organics associated with blooms. Previous studies have demonstrated that bloom conditions are associated with an increase in DBP pre-cursors and DBPs (Zamyadi et al., 2012; Zong et al., 2015; Foreman et al., 2021). Disinfectants and chlorine react easily with all types of organic matter, including dissolved and algal organic matter to produce genotoxic and carcinogenic DBPs including trihalomethanes (THMs) and haloacetic acids (HAAs) (Liu et al., 2018). Therefore, conventional water treatment using activated carbon can be useful for removing cyanotoxins, and dissolved and algal organic compounds (Ho et al., 2011; U.S. EPA, 2016; Cermakova et al., 2017).

While activated carbon and rest procedures reduce unwanted contaminants, biofilms can become established in the system and contribute to promoting antibiotic resistance genes (ARGs) (Guo et al., 2018). Specifically, activated carbon filtration has been linked to increasing the abundance of antibiotic resistance bacteria (ARB) and antibiotic resistance genes (ARGs) (Xu et al., 2016, 2020a). Furthermore, recent studies report that cyanobacteria, especially *Microcystis*, harbor significant amounts of ARGs and may play a significant role as a reservoir and source for ARGs in bloom seasons whereby cyanobacterial blooms promote conjugative transfer of ARGs (Wang et al., 2020, 2021). More studies recently reported that cyanobacteria may promote the spread of ARGs in bacteria due to the significant contribution of mobile genetic elements (MGEs) located in genera, such as *Microcystis* (Volk and Lee, 2022). The interest in *Legionella* spp. is heightened in bloom-affected source waters as biofilm-loving *Legionella* spp. including *L. pneumophila* have long been known to grow when cyanobacteria densities are great and biofilm masses exist (Tison et al., 1980; Fliermans et al., 1981; Pope et al., 1982; Declerck, 2010; Cai et al., 2014). Additionally, biofilms can support the growth and persistence of *Legionella* spp. in drinking water systems (Berjeaud et al., 2016) including where activated carbon is used (Li et al., 2017).

While drinking water treatment plants (DWTPs) in bloom-affected areas give necessary and considerable attention to removing cyanotoxins and cyanobacteria during bloom seasons, there are emerging issues not yet studied in the Lake Erie region. We hypothesize that water from heavy bloom areas have (1) higher DBP levels in finished water due to increased interactions between bloom-associated organic matter and chlorine during treatment, (2) higher ARG from using activated carbon to absorb toxins as well as from the higher abundance of *Microcystis* in the source water, and (3) bloom-related aggregated biomass provides a niche for promoting *Legionella* in the lake water and is thus positively associated with *Microcystis* in Lake Erie. In the present study, we investigated two water treatment plants in the Lake Erie region (western Lake Erie for greater bloom frequency and intensity, and central Lake Erie for low bloom frequency and intensity) for 2 years. Specifically, we (1) examined the dynamics of *Microcystis* and MCs in the source water, (2) determined the association between cyanobacteria and *Legionella* in the source water, and (3) monitored both DBPs and ARGs in their finished water. The study outcome provides more holistic understanding about the water quality issues beyond bloom toxicity and cyanotoxin where cyanobacterial blooms are chronically prevalent.

## Materials and methods

### Study sites and water samples

Two drinking water treatment plants in Ohio, United States, were selected based on annual cyanobacterial bloom intensity. The first was the Collins Park Water Treatment Plant in Toledo, which is a historically high-bloom area in western Lake Erie (Ohio Department Health et al., 2020) and the second was the City of Painesville Water Treatment Plant, a low- or no-bloom area in central Lake Erie (Supplementary Figure S1). Water samples were collected once weekly during the summer and fall (May–November) of 2013 and 2014 from the Toledo and Painesville sites, totaling 58 and 60 samples, respectively. Samples were collected from the source water (sampled at the drinking water intakes within Lake Erie) and from the finished drinking water at each plant. Collections were done in sterile bottles and shipped in a cooler on ice. For toxin measurements, amber glass vials were used. The samples reached The Ohio State University (Columbus, Ohio, United States) for laboratory analysis within 15 h of sample collection. In the lab, the collected source water (200 mL) was filtered using the polycarbonate membrane (0.45 μm pore size, Millipore, Burlington, MA, United States) for molecular analyses, and filters were kept at −20°C and further analyses were performed as soon as possible.

### Water quality parameters

Conventional water quality parameters (water temperature, turbidity, hardness, and pH) and total chlorine in finished water were measured routinely on-site according to the Standard Methods (Rice et al., 2012) by the two water treatment plants and the data were obtained. Chlorophyll-*a* and phycocyanin were measured using the AquaFluor^®^ Handheld fluorometer (Turner Designs, California, United States). Total phosphorus (TP) and soluble reactive phosphorus were measured using the USEPA-accepted Method 8190 (Hach PhosVer 3 with Acid Persulfate Digestion). Total nitrogen (TN) in the higher range (0.23–13.50 mg/L NO_3_^−^-N) and the lower range (0.01–0.5 mg/L NO_3_^−^-N) were analyzed using Method 10206 (Hach dimethylphenol method) and Method 8192, respectively. Total organic carbon (TOC) was analyzed at Alloway Laboratory (Marion, Ohio, United States) using the USEPA 451.3 (U.S. EPA, 2005).

### MC measurement

Total MCs in water samples were measured using enzyme-linked immunosorbent assay (ELISA) kits (Abraxis, Warminster, PA, United States) with MC-LR (0.15–5 μg/L) as a working standard. The absorbance was read at 450 nm on the Dynex MRX microplate reader (Dynex Technologies. Inc., Chantilly, VA, United States). The detection range of the assay is 0.15–5.0 ng/mL with a detection limit of 0.10 ng/mL. All the ELISA assays were performed in triplicate.

### Disinfection by-products

DBPs (TTHMs and HAA5) in finished water were quantified with gas chromatography–mass spectrometry (GC–MS) using the USEPA-524.2 method.^1^ TTHMs included bromoform (CHBr_3_), chloroform (CHCl_3_), bromodichloromethane (CHCl_2_Br), chlorodibromomethane (CHClBr_2_). HAA5 included monchloroacetic acid (CH_2_ClCOOH, MCAA), dichloroacetic acid (CHCl_2_COOH, DCAA), trichloracetic acid (CCl_3_COOH, TCAA), monobromoacetic acid (CH_2_BrCOOH, MBAA) and dibromoacetic acid (CHBr_2_COOH, DBAA). DBPs were quantified at Alloway Laboratory (Marion, Ohio, United States).

### Quantification of total and toxic *Microcystis* abundance

We quantified concentrations of total and MC-producing *Microcystis* by targeting the *Microcystis* phycocyanin intergenic spacer (PC-IGS) and microcystin synthetase gene B (*mcyB*), using published approaches with slight modifications (Klase et al., 2019). For *Microcystis* PC-IGS, the PCR mixture (20 μL) in duplicate contained TaqMan universal PCR master mix (10 μL) (Life Technologies, United States), each primer (188F/254R, 300 nM), PC-IGS probe (100 nM), additional MgCl_2_ (1.25 mM) and DNA template (2 μL). The PCR profile followed an initial cycle of 50°C for 2 min, 95°C for 10 min, 50 cycles at 95°C for 30 s, 56°C for 1 min, and 72°C for 30 s. For *Microcystis mcyB*, the PCR protocol was identical to that for PC-IGS, except we used 900 nM of each *mcyB*-specific-primer (30F/108R) and 250 nm of *mcyB* probe. The standard working curve was generated for each assay according to the standard *Microcystis* PC-IGS and *mcyB* DNA standard (pGEMT-PC-IGS and *mcyB*), respectively. The PC-IGS probe had a 5′ end of the fluorescent reporter, 6FAM, and a 3′ end of the non-fluorescent quencher (NFQ) that was attached with a minor groove binder (MGB) moiety (MGB-NFQ). In contrast, the *mcyB* probe contained a 5′ end of the fluorescent reporter, instead. We estimated the proportion of the potentially MC-producing genotype in the *Microcystis* population according to the ratio between *Microcystis mcyB* and PC-IGS in percentage unit (%).

### Droplet digital PCR for total bacteria, ARGs and *Legionella*

We targeted three antibiotic resistance genes (*tetQ* for tetracycline resistance, *sul1* for sulfonamide resistance, and *bla*_KPC_ for carbapenem resistance), total bacteria (targeting 16S rRNA gene), *Legionella* species (targeting the 5S rRNA gene), as well as a mobile genetic element [MGE, class 1 integron-integrase gene (*intI1*)] (Krøjgaard et al., 2011; Jia et al., 2014; Yin et al., 2017; Gupta et al., 2019; Klase et al., 2019) using the QX200 droplet digital PCR system (ddPCR, Bio-Rad, Hercules, CA, United States). For quantification of total bacteria, *tetQ*, *sul1*, and *intI1*, the ddPCR mixture (20 μL) contained 2X EvaGreen supermix (Bio-Rad), 200 nM of each primer, DNA template, and RNase-/DNase frees PCR water. To quantify KPC and *Legionella*, the ddPCR mixture (20 μL) contained 2X supermix for probes, 200 nM of each primer, 200 nM of the probe, DNA template, and RNase-/DNase free PCR water. With 20 μL of PCR mixture, droplets were generated using the Droplet generator (Bio-Rad) with droplet generation oil. PCR was performed using a thermal cycler (C1000 touch thermal cycler, Bio-Rad, Hercules, CA, United States), following previous studies (Yin et al., 2017; Gupta et al., 2019; Klase et al., 2019). After the PCR reaction, concentrations of targeted genes were analyzed using the Droplet Reader and QuantaSoft software (Bio-Rad Hercules, CA, United States).

### Statistical analysis

Data were explored using scatterplots, time-series plots, box plots and bar charts. Means and standard deviations (SD) were calculated and data are presented as the mean ± SD. A one-way analysis of variance (ANOVA) was used for the spatial difference in the variables with a significance level set at *p* < 0.05. After ANOVA, Tukey Honest Significant Difference tests were used to determine if the means were different (*p* < 0.05) between the groups. All analyses were conducted using SPSS 24.0 (SPSS, Chicago, IL, United States).

## Results

### Source water

**Figure 1:**
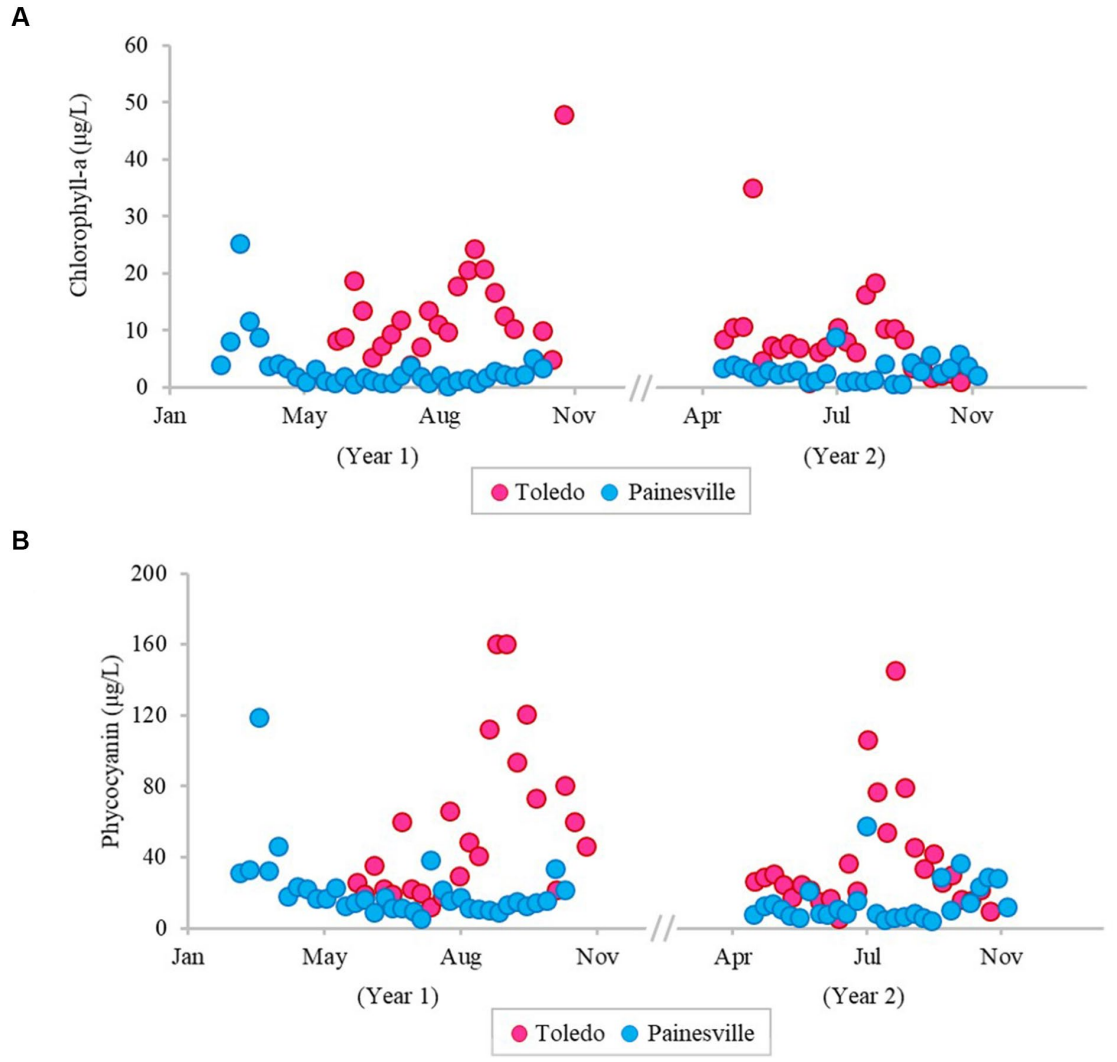
Temporal changes of concentration (pg/L) of bloom-related parameters, chlorophyll-a **(A)** and phycocyanin **(B)**, in source water from the bloom site (Toledo) (dark pink) and the control site (Painesville) (blue).

**Figure 2:**
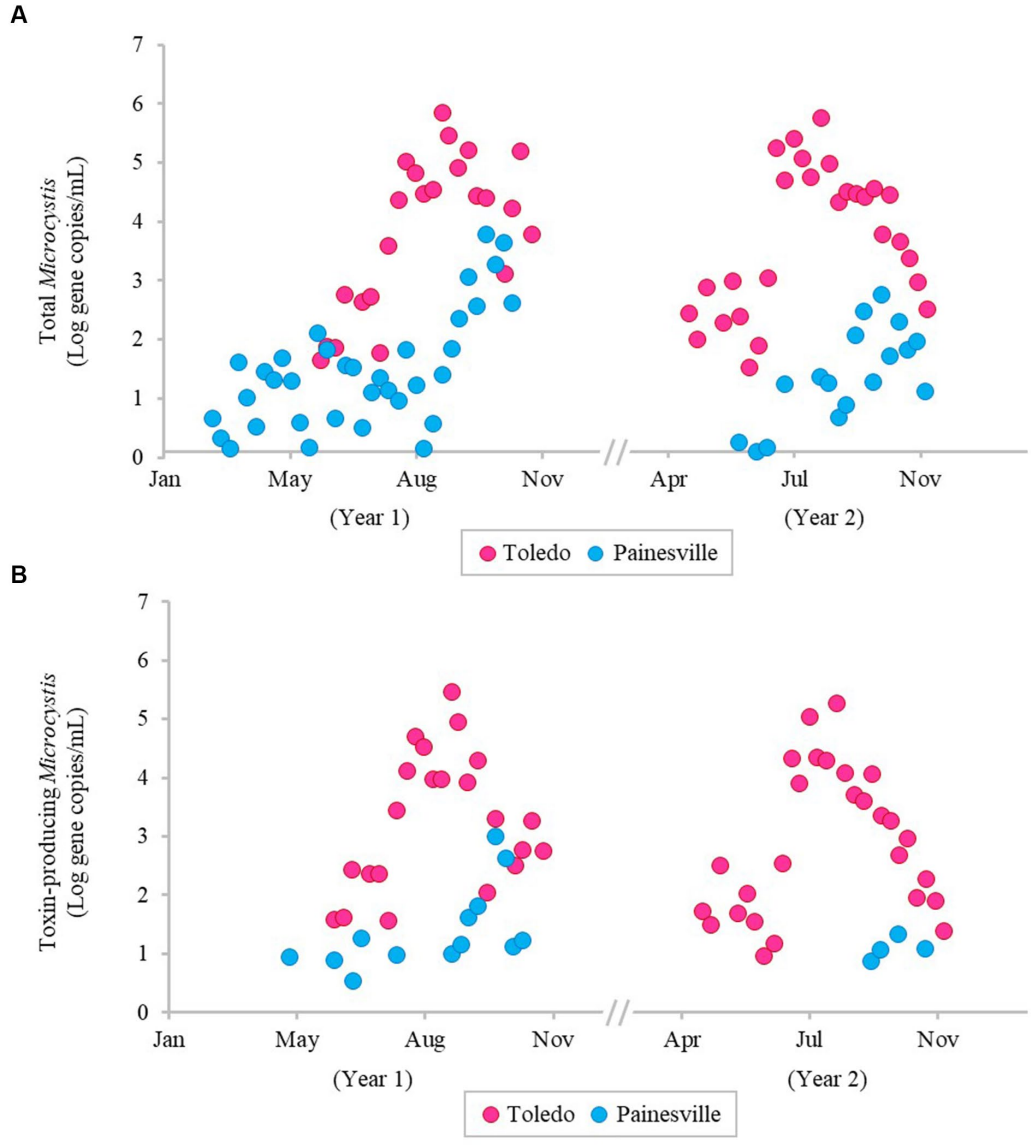
Temporal changes of population of total Microcystis **(A)** and toxin-producing Microcystis **(B)** in source water from the bloom site (Toledo) (dark pink) and the control site (Painesville) (blue).

**Figure 3:**
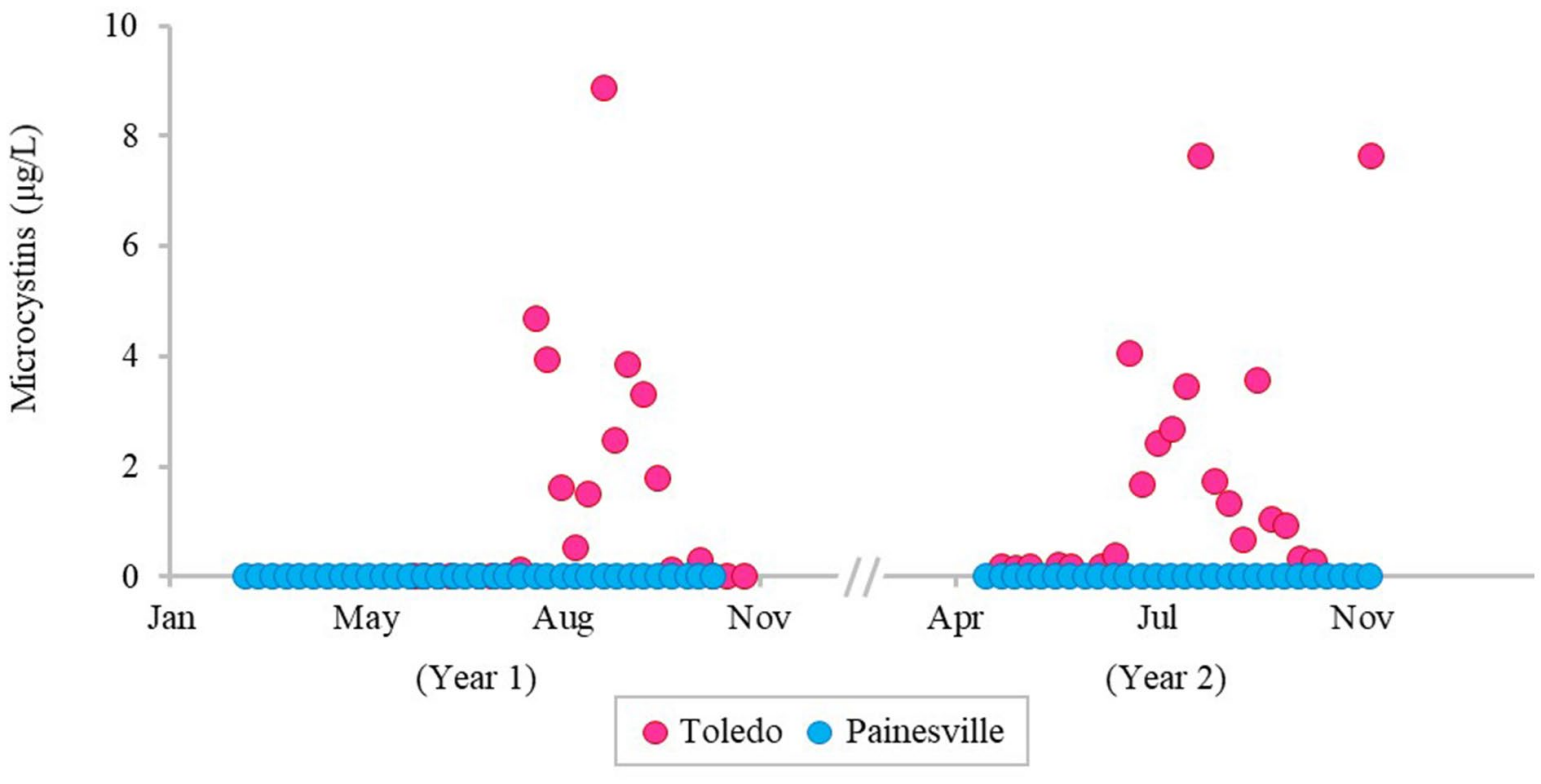
Temporal changes of concentration of microcystin in source water from the bloom site (Toledo) (dark pink) and the control site (Painesville) (blue).

The severity of blooms in the source water from western (Toledo) and central (Painesville) Lake Erie was examined (Figures 1–3) as were source water chemical parameters and water temperatures. The mean concentrations of chlorophyll-*a* and phycocyanin in the source water from western Lake Erie (bloom site) were 8.32 μg/L and 37.4 μg/L, respectively (Figure 1). The mean concentrations of chlorophyll-*a* and phycocyanin in the source water from central Lake Erie (control site) were 2.92 μg/L and 14.8 μg/L, respectively (Figure 1). The mean concentrations of two bloom indicators in the bloom site were significantly higher than that from the control site (*p* < 0.05). Similarly, significantly higher concentrations of total *Microcystis* (PC-IGS gene) and toxin-producing *Microcystis* (*mcy*B gene) were observed in the bloom site source water (*p* < 0.05) (Figure 2). Specifically, the mean concentration of the PC-IGS gene (gene copies/mL) was 5.57 × 10^4^ in Toledo source water (bloom site) vs. 8.79 × 10^1^ in the Painesville source water (control site). The mean concentration of the *mcy*B gene (gene copies/mL) was 1.48 × 10^4^ in the bloom site vs. 1.32 × 10^1^ in the control site. In regard to the cyanotoxin measurement, the mean concentration of microcystin in the bloom site source water was 1.65 μg/L which was also higher than the control site (not detected) (Figure 3). These results show that the intensity and frequency of blooms at the bloom site (Toledo) was obviously higher than the control site (Painesville).

Among water chemistry parameters, concentrations were consistently higher at the bloom sites; whereby mean total phosphorus levels for the two-year study period were 140 μg/L and 90 μg/L for the Toledo and Painesville DWTP source water samples. The mean total organic carbon was 0.36 mg/L (Toledo) and 0.24 mg/L (Painesville) and the mean total nitrate was 0.64 mg/L and 0.33 mg/L in the Toledo and Painesville source water samples, respectively. In addition to chemistry parameters, the mean temperatures of source water samples were 19.6°C and 16.1°C for Toledo and Painesville, respectively.

**Figure 4:**
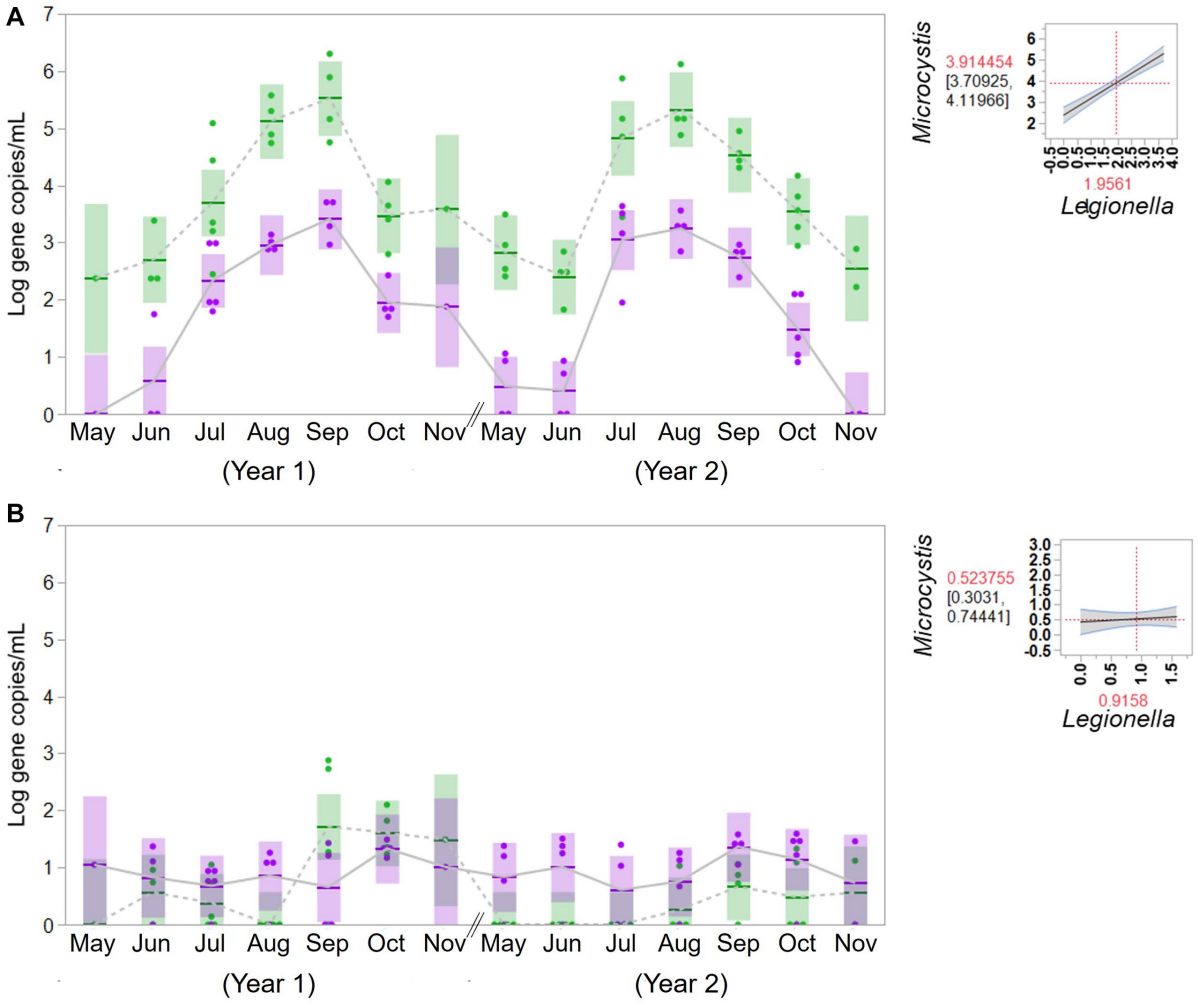
Temporal change of concentration of Microcystis (green) and Legionella (purple) in source water from the bloom site (western Lake Erie, Toledo) **(A)** and the control site (central Lake Erie, Painesville) **(B)**. Statistical analysis for relationship between total cyanobacteria and Legionella species in the source water.

To evaluate the potential association between cyanobacteria and *Legionella*, the concentrations of *Legionella* were examined in the source water from the western Lake Erie and the control site for 2 years. The concentrations of *Legionella* (gene copies/mL) were 2.49 × 10^2^ (ranging from 5.10 × 10^0^ to 5.09 × 10^3^) in the bloom source water and 1.63 × 10^2^ (ranging from 4.66 × 10^1^ to 3.85 × 10^2^) in the control source water. The associations between cyanobacteria and *Legionella* in the source water are summarized in Figure 4. An apparent significant relationship was observed in western Lake Erie between the concentration of the molecular markers for total cyanobacteria and *Legionella* spp. (*F* = 89.82, *p* = 0.001), but there was no relationship observed between the markers for total cyanobacteria and *Legionella* spp. observed in source water samples from the non-bloom site in Painesville (*F* = 0.31, *p* = 0.5814).

### Finished water

**Figure 5:**
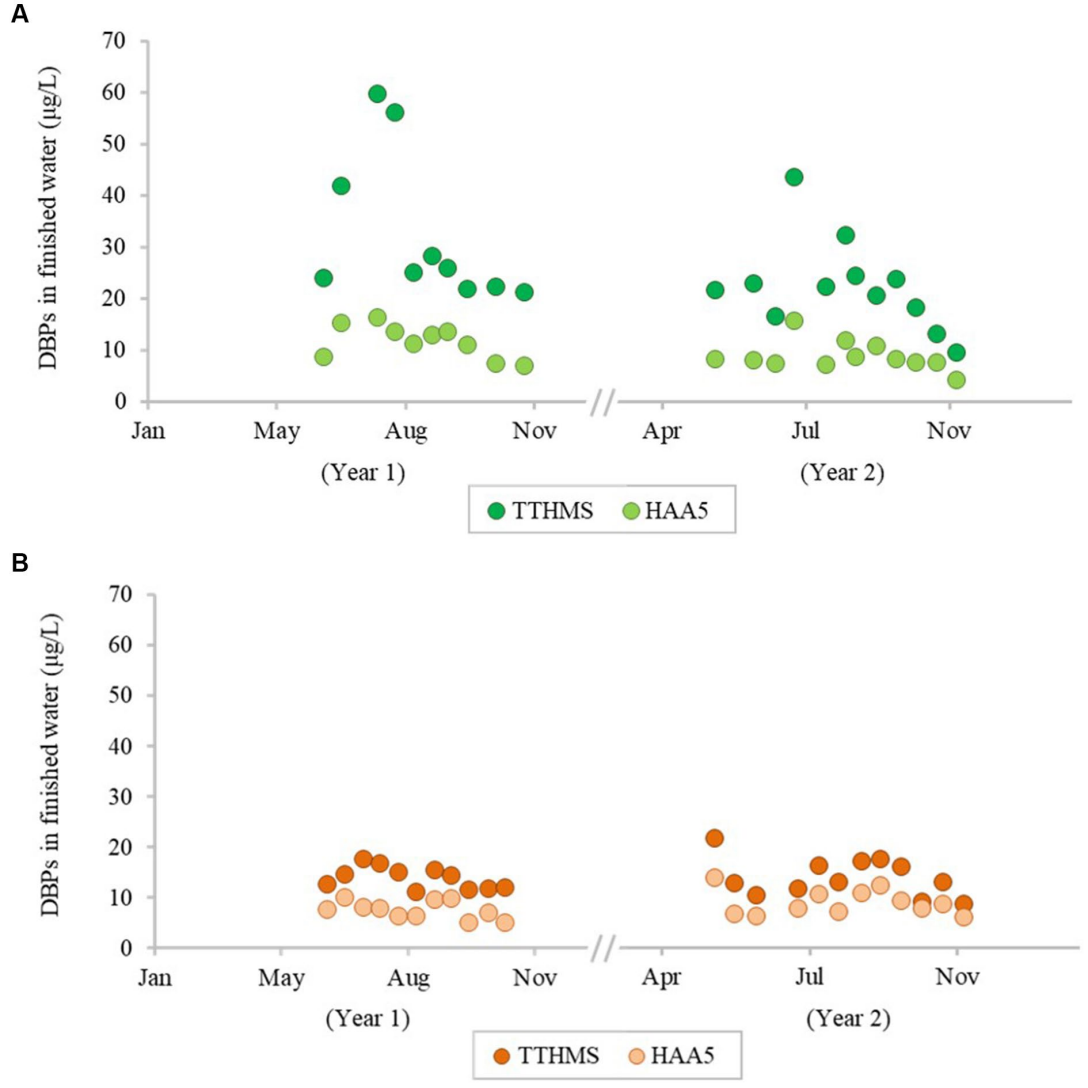
Temporal change of concentration of disinfection by-products in finished water from the bloom site **(A)** and the control site **(B)**.

**Figure 6:**
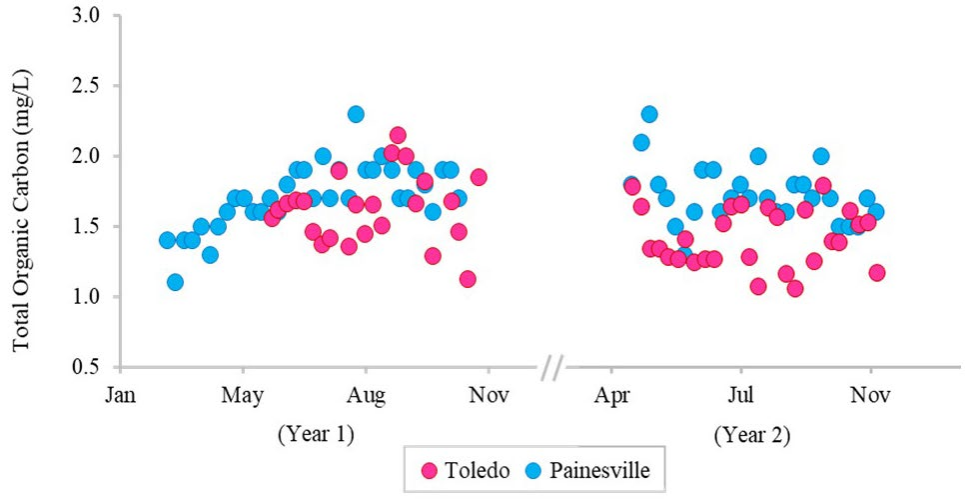
Temporal change of concentration of total organic carbon in the finished water of the bloom site (Toledo) (dark pink) and the control site (Painesville) (blue).

The concentrations of DBPs (TTHMs and HAA5) in the finished water from the bloom vs. control sites are summarized in Figure 5. The mean concentrations of TTHMs were 22.43 μg/L from the bloom site and 14.03 μg/L from the control site, and the mean TTHMs concentration from the bloom site was significantly higher than the control site (*p* < 0.05). The mean concentrations of HAA5 were 8.86 μg/L from the bloom site and 9.06 μg/L from the control site, and the mean HAA5 concentrations between the two locations were not significantly different (*p* > 0.05). Figure 6 shows that TOC levels in finished water were not statistically different between the bloom vs. control sites (*p* > 0.05). The mean concentrations of TOC were 1.42 mg/L from the bloom site and 1.72 mg/L from the control site. Furthermore, microcystin was not detected in finished water samples from either location.

**Figure 7:**
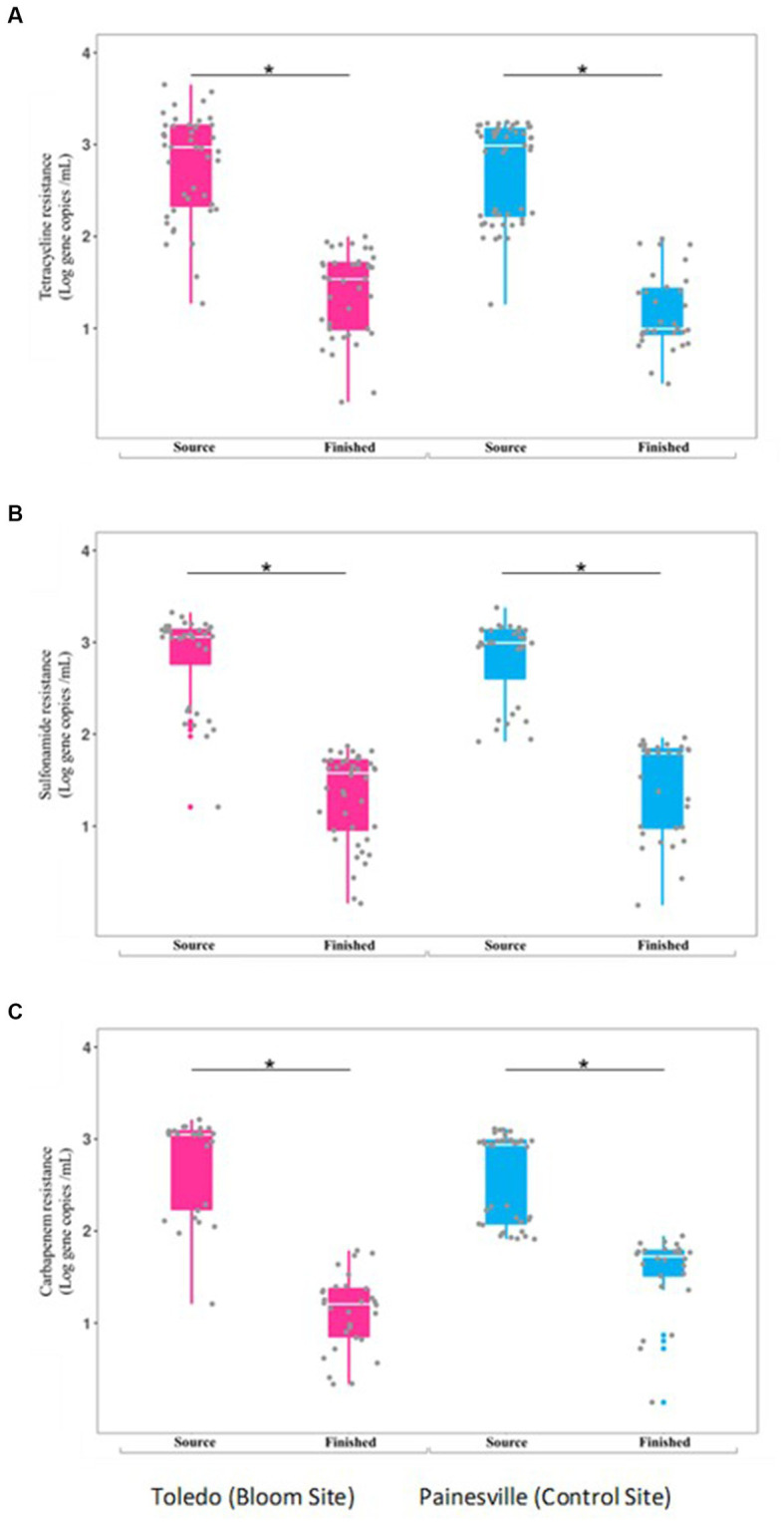
Concentrations of antibiotic resistance genes [tetQ **(A)**, sull **(B)**, and blak PC **(C)**] in source and finished waters from the bloom site (Toledo) (dark pink) and the control site (Painesville) (blue). *p < 0.05.

Concentrations of ARGs [tetracycline (*tetQ*), sulfonamide (*sul1*), carbapenem (*bla*_KPC_) resistance genes] were quantified in source and finished waters. The concentrations of all the ARGs were significantly lower (*p* < 0.05) in the finished water vs. the source water at bloom and control locations (Figure 7). In comparing the mean concentrations (gene copies/100 mL) of ARGs in the finished water, the Toledo finished water mean ARG concentrations were 3.70 × 10^2^ (*tetQ*), 3.26 × 10^2^ (*sul1*), and 1.91 × 10^2^ (*bla*_KPC_). The mean concentrations (gene copies/100 mL) of ARGs in the finished water from Painesville WTP were 2.43 × 10^2^ (*tetQ*), 4.31 × 10^2^ (*sul1*), and 4.69 × 10^2^ (*bla*_KPC_).

**Figure 8:**
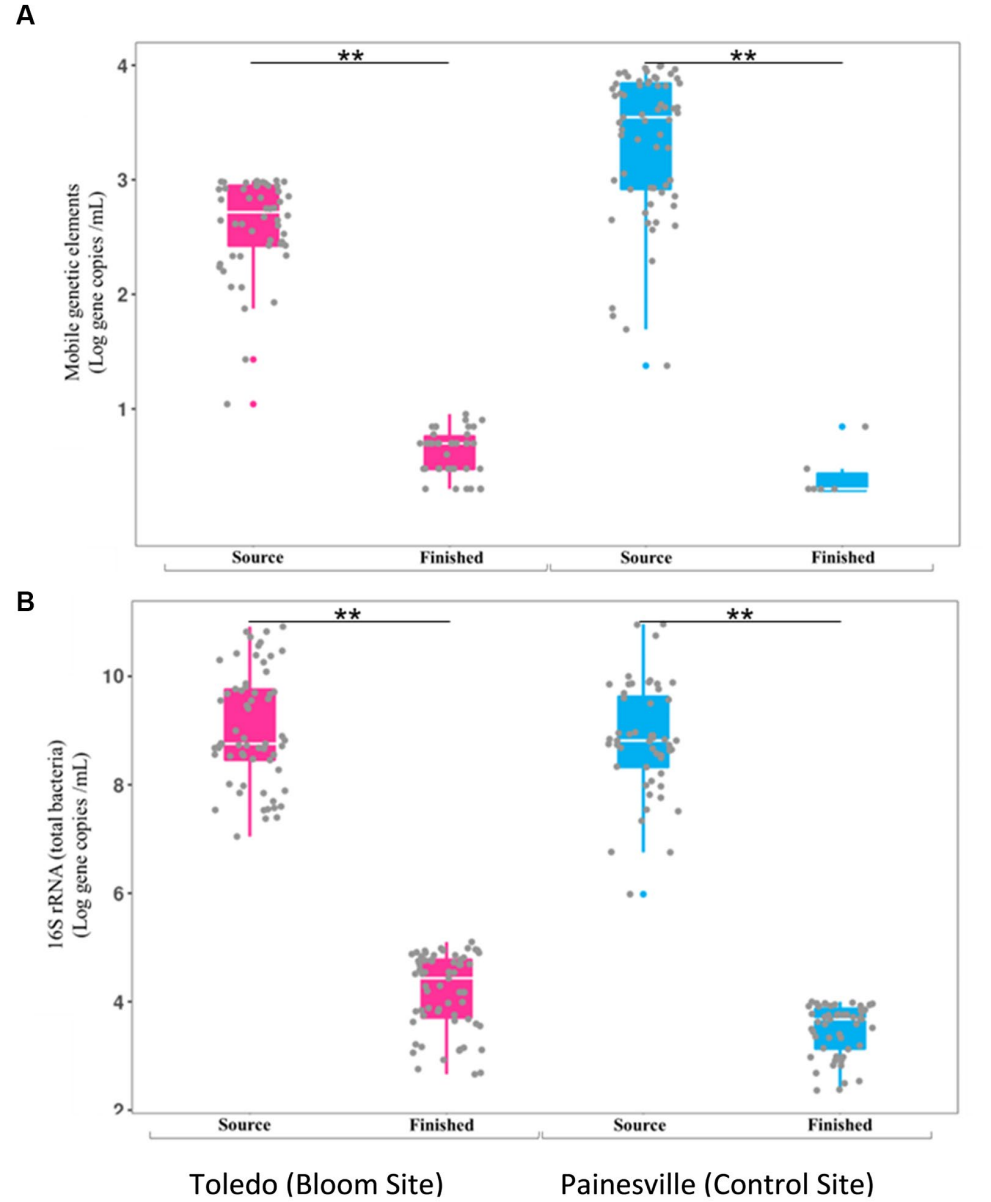
Concentrations of mobile genetic elements [MGES **(A)**] and 16S rRNA [total bacteria **(B)**] in source and finished waters from the bloom site (Toledo) (dark pink) and the control site (Painesville) (blue). **p < 0.01.

**Figure 9:**
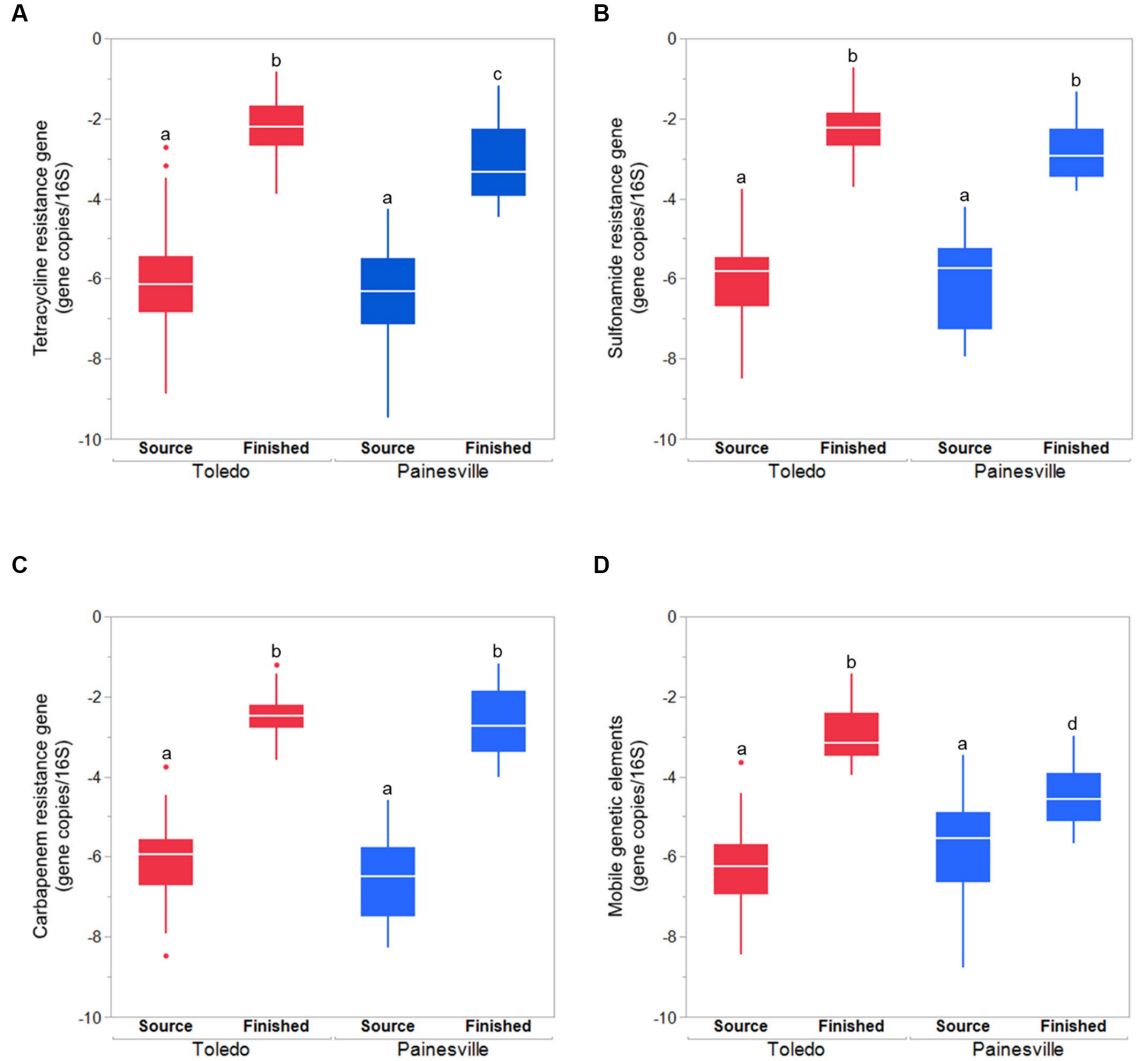
The relative abundances (gene copies/16S rRNA gene) of ARGS [tetracycline **(A)**, sulfonamide **(B)**, carbapenem resistance genes **(C)**] and mobile genetic element **(D)** in source water and finished water from the bloom site (Toledo) and the control site (Painesville). ^b^Represents a significant difference from “a” (*p* < 0.01). ^c^Represents a significant difference from “a” (*p* < 0.01) and from “b” (*p* < 0.05).

Concentrations of MGE (class 1 integron-integrase gene [*intI1*]) and the total bacteria gene (16S rRNA) were also significantly reduced by drinking water treatment. After water treatment processing, the mean concentrations of the MGE (gene copies/100 mL of *intI1*) in the finished water were 4.19 × 10^0^ in Toledo and 0.82 × 10^0^ in Painesville which represented significant (*p* < 0.01) reductions from their source water (Figure 8). Similarly, concentrations of the total bacterial gene in the finished water of the bloom and control sites (4.56 × 10^3^ in Toledo, 3.55 × 10^4^ in Painesville) were significantly lower (*p* < 0.01) than in their source water (6.48 × 10^8^ in Toledo, 9.00 × 10^9^ in Painesville). While drinking water treatment resulted in significantly lower concentrations of total bacteria, ARGs, and MGE, the relative abundance of ARGs among total bacteria (16S rRNA) increased significantly (*p* < 0.05) at both the bloom and control sites (Figure 9).

## Discussion

### Bloom conditions at the western Lake Erie site

Two drinking water treatment plants, drawing from Lake Erie as their source water, were chosen in Toledo (a high-bloom region in western Lake Erie) and Painesville (a low- or no-bloom area in central Lake Erie) to examine water quality challenges beyond cyanotoxins when utilizing bloom-impacted source water. In assessing blooms, with an emphasis on cyanobacteria, multiple water quality parameters were employed to evaluate source and finished water quality. Upon characterizing bloom conditions, as expected, there were significant differences in chlorophyll-*a*, phycocyanin, total *Microcystis*, MC-producing *Microcystis*, and MC between the two locations. These differences were supported by the underlying water chemistry and temperatures enabling greater primary productivity by cyanobacteria as warmer water temperatures and higher concentrations of nutrients (P and N) are linked to cyanobacteria bloom formation, particularly when water temperatures approach or exceeds 20°C (Paerl and Huisman, 2008; Stumpf et al., 2012).

As predicted, bloom conditions manifested in source water during the warm months, mainly from July, August and September at the Toledo water intake (bloom site). Figures 1–4 show corroboration between bloom indicators (chlorophyll-*a* and phycocyanin), *Microcystis* densities, and ultimately cyanotoxins. Three of the source water MC results from Toledo exceeded the provisional 2015 Ohio contact recreation advisory threshold (6 μg/L) and one sample exceeded the revised and current (post-2019) Ohio and U.S. EPA contact recreation advisory threshold (8 μg/L standard) (Kasich et al., 2015; U.S. EPA, 2019). Notably, although MC levels were elevated in Toledo’s source water, they remained undetectable in finished water samples. Of note, the *Microcystis* concentrations indicated by PC-IGS genetic marker measurement in the source water in Toledo were similar to those in a Japanese Lake Mikata where *Microcystis* blooms occurred (Yoshida et al., 2007).

### Elevated DBP formation potential and DBPs from bloom conditions

Once cyanobacterial blooms occur, blooms can impair surface water supplies used for drinking water. Blooms are associated with increases in TOC, turbidity, taste and odor compounds, and precursors for DBP formation (Nguyen et al., 2005). As hypothesized, TOC and turbidity levels were elevated in the bloom site source water relative to the control site (Painesville). Algal-derived organic carbon can be a significant source of DBP precursors for drinking water treatment facilities (Nguyen et al., 2005).

While DBP precursors can be elevated in source water, the presence of DBPs in finished drinking water varies according to the source water characteristics (e.g., temperature, pH, natural organic matter, etc.) and the processes used in water treatment. TOC concentrations are often used to predict DBP formation because TOC is a precursor to DBP formation. In this study, we investigated not only temporal variation in TOC (DBP formation precursor), but also the concentration of two commonly regulated DBPs, TTHMs and HAA5, in drinking water. TTHMs and HAA5, considered potentially carcinogenic, are the most important groups of DBPs (Rodriguez et al., 2004). The primary drinking water regulations for the U.S. mandates that the maximum acceptable levels of DBPs are 80 μg/L for TTHMs and 60 μg/L for HAA5 (U.S. EPA, 2023). Our results show that TOC concentrations in Toledo source water were higher than Painesville source water, and it is likely that the increase in TOC is related to cyanobacteria densities (Cory et al., 2016). As TOC levels were higher in the source water at Toledo, so were TTHMs and HAA5 in the finished water relative to the Painesville finished water. While DBPs were quantifiable, none of the samples exceeded primary standards set by the U.S. EPA, indicating that the current TOC removal processes at the two DWTPs can effectively control common DBPs.

### Relationship between *Legionella* and *Microcystis* in bloom site source water

Beyond altering source and finished water quality, cyanobacteria blooms in western Lake Erie occur in the context of a complex microbial ecosystem and can alter microbial communities.

One type of change was the observed apparent positive relationship between *Legionella* spp. abundance and *Microcystis* in the source water for the Toledo DWTP (bloom site), which was present in both study years, but non-existent in the source water of the Painesville DWTP that served as a control site. Recent research from western Lake Erie (near Toledo [bloom site]) focusing on bacterial diversity from non-cyanobacteria showed clear differential responses among non-cyanobacteria to cyanobacteria abundance (estimated by chlorophyll-*a*) (Berry et al., 2017). Among bacteria that are linked to an increase in abundance during bloom conditions, there is evidence that the density of *Legionella* is correlated with *Microcystis* and eutrophication (Numberger et al., 2022). When looking at similar warm season water samples from three inland Ohio lakes, which like Western Lake Erie exhibit eutrophic conditions, Lee et al. (2016) documented *Legionella* spp. and the Legionellacea as being among the most abundant and ubiquitous bacteria present in Ohio inland lakes.

While *Legionella* species are common in various natural and human-made aquatic environments, some species, mostly *L. pneumophila*, may cause legionellosis, which is a serious pulmonary infection established in persons following the inhalation of particles of contaminated aerosolized water. While there is some evidence that certain cyanobacteria can stimulate the growth of *Legionella* (Świątecki and Zdanowski, 2007; Bergeron et al., 2015), there are few studies primarily focusing on *Legionella* and cyanobacteria densities. Tison et al. (1980) suggested an intimate association between *Legionella* spp. and cyanobacteria (*Fisherella*). Their observation indicated that *Legionella* could use algal extracellular products as its carbon and energy sources (Tison et al., 1980; Berendt, 1981). A previous study also reported that symbiotic interactions between *Legionella* and cyanobacteria may help the colonization of aquatic environments (Carvalho et al., 2007). Several factors (e.g., temperature, pH, and concentrations of nutrients and ions) and possibly products produced by other non-cyanobacterial bacteria, such as earlier arriving Betaproteobacteriales (van der Kooij et al., 2018), which are also abundant in Lake Erie during blooms (Berry et al., 2017), may all contribute to *Legionella* growth in aquatic environments as part of an ecological succession linked to bloom conditions of certain cyanobacteria species, like *Microcystis*.

While the potential for an ongoing increased global incidence of legionellosis due to a warmer climate has been described (Walker, 2018), the role of environmental waters warrants additional study. In Ohio, the incidence rate of legionellosis has consistently been 1.9 to 2.6 times greater than the U.S. average in recent years (Han, 2021). Recently, aerosols associated with roadway exposures have been considered as part of the increased incidence (Han, 2021), but of particular interest here is how anticipated future increases in eutrophic waters experiencing further eutrophication and warming globally may contribute to increased aerosol-related *Legionella* exposures, which could occur from boat wake (water skiing), fountains, etc. Beyond warmer water, there may be possible synergisms occurring in warming eutrophic waters as high concentrations of phosphates can enable substantial growth of cyanobacteria and increased turbidity and/or aggregated biofilm materials which can prompt or enhance the growth of *Legionella* and other Gammaproteobacteria (Cai et al., 2013; Lee et al., 2016; Srivastava et al., 2016). While the Gammaproteobacteria include many biofilm formers that may benefit from increased turbidity, these bacteria are also associated with an abundance of antibiotic resistance genes (Zhang et al., 2021).

### Relative abundance of ARGs increases in finished water from Lake Erie

Adding to the complexity of bloom ecology beyond *Legionella* densities are other broader emergent concerns regarding the establishment, maintenance, or promotion of antibiotic resistance bacteria (ARB) and ARGs in the source water (Volk and Lee, 2022), finished water (Li et al., 2016), and the distribution system (Xi et al., 2009; Xu et al., 2016). When the source water is impacted by bloom conditions, a convergence of numerous selective pressures emerge in the microbial community, including interspecies competition in the source water coupled with impacts from the water treatment processes, such as activated carbon use and disinfection. Xu et al. (2020b) demonstrated that when MCs were present an increase in ARGs was observed in the DWTP.

In this study, while hypothesized there would be differences in the densities of MGEs and ARGs in the source water samples from bloom and control sites, no differences were observed for MGEs or the ARGs for tetracycline, sulfonamide, and carbapenem resistance (Figure 7). At both study locations, the DWTP processes significantly reduced the amount of MGEs and ARGs from source water to finished water. The differences that emerged were specific to relative abundances of ARGs and MGEs. At both DWTPs, while reduced in overall density, the total bacterial density was reduced even more greatly by the treatment processes. In comparison of the two DWTPs, the MGEs increased in relative abundance more in the Toledo DWTP than the Painesville DWTP.

Previous studies have revealed that commonly used disinfection technology can enrich ARB and spread ARGs (Shi et al., 2013). Water processing, including filtration and chlorination, remove most bacteria; however, extracellular stress can promote the replication of plasmids in bacteria. For example, chlorination might increase the copy number of plasmids in the surviving bacteria cells, resulting in the higher relative abundance of ARGs in treated water (Shi et al., 2013). While densities of ARGs and MGE were higher in the source water than the finished water, a limitation of this study was that it did not study the water distribution system, which can have higher levels of bacteria than the finished water due to regrowth potential of bacteria in the distribution system (Xi et al., 2009). If the source water selected for greater survival of biofilm formers preferring higher phosphate levels, corrosion control measures for the distribution system using phosphate may play a role in the regrowth of antibiotic resistant bacteria (Kappell et al., 2019; Kimbell et al., 2020). As expected, the finished water which included corrosion control had higher total P levels than the source water at the bloom site and control site (Supplementary Table S1).

### Future research needs

The main scope of the present study was source water and finished water in Lake Erie region, thus tap samples or samples from within the distribution system were not included in this study, but we suggest that future study includes examining the distribution systems of bloom-impacted community water systems since it can contribute great knowledge regarding the public health implications of biofilm formation, ARGs, MGEs, and potential bacterial regrowth. This study adds to the body of evidence that some efforts should be made to monitor ARG concentrations before, during, and after drinking water treatments. As part of monitoring for ARGs and *Legionella*, methods reliant on culturable *Legionella* or other bacteria (e.g., *Pseudomonas* spp., *Mycobacterium* spp.) may provide benefits, but may underreport densities of some bacteria resistant to disinfection when hosted inside free-living Amoeba, which warrants a need to use a PCR-based approach (Calvo et al., 2013).

Complicating future studies on ARGs and MGEs in finished water and distribution systems are not only disinfectants but also disinfection byproducts. While this study illustrated that chlorine-related DBPs (THMs and HAA5) were elevated during bloom conditions, yet meeting regulatory thresholds, we suggest to characterize other DBPs that are likely elevated in cyanobacteria-impacted waters. Since there is considerable evidence that THMs and HAAs are linked to ARGs in the finished water and distribution system (Lv et al., 2014; Li and Gu, 2019; Zhang et al., 2023), we recommend to assess unregulated N-DBPs which likely are less abundant than THMs and HAAs, but are more common in bloom-impacted waters, and able to elicit greater cytotoxicity (Fang et al., 2010; Liu et al., 2020) in a future study. In addition, emerging literature indicates that future studies on DBPs ought to consider a broader range of DBPs for also assessing potential human health risks (Li and Mitch, 2018; Kali et al., 2021). Thus, in bloom-impacted waters there would likely be benefits from assessing N-DBPs which are associated with the chlorination of *Microcystis aeruginosa* (Fang et al., 2010). These same N-DBPs may also be of interest for improving understanding of antibiotic resistance phenomena in treated waters.

## Conclusion

In this study, a compelling and statistically significant correlation emerged between *Legionella* and cyanobacteria within the water of the bloom site. Increasing levels of *Microcystis* were also associated with disinfection byproducts (THMs and HAA5) in the water of the bloom site, but did not exceed primary regulatory standards. At both the bloom site and control site, the DWTPs reduced the density of ARGs and MGEs; however, their relative abundance increased in finished water. This study not only fills existing gaps in the understanding of cyanobacteria and *Legionella* ecology but also underscores several pivotal areas of needed future research for aquatic environments with human exposure potential. Furthermore, our findings indicate a potential health risk of *Legionella*-related disease in proximity to areas impacted by blooms.

## Data availability statement

The original contributions presented in the study are included in the article/Supplementary material, further inquiries can be directed to the corresponding author.

## Author contributions

JL: conceptualization, funding acquisition, methodology, supervision, and writing-original draft. SL: formal analysis, methodology, visualization, writing—review and editing. CH: formal analysis, methodology, visualization, and writing—review and editing. JM: methodology, writing-review and editing. All authors contributed to the article and approved the submitted version.

## Funding

This study was partially funded by US EPA Science to Achieve Results (STAR) grant (RD835192010).

## Acknowledgments

The assistance provided by drinking water treatment plants in Toledo and Painesville, Ohio, and their staff members are greatly appreciated. The authors are thankful for Tyler Gorham for his help in processing water samples.

## Conflict of interest

The authors declare that the research was conducted in the absence of any commercial or financial relationships that could be construed as a potential conflict of interest.

## Publisher’s note

All claims expressed in this article are solely those of the authors and do not necessarily represent those of their affiliated organizations, or those of the publisher, the editors and the reviewers. Any product that may be evaluated in this article, or claim that may be made by its manufacturer, is not guaranteed or endorsed by the publisher.

## Footnotes

^1^ https://www.epa.gov/esam/epa-method-5242-measurement-purgeable-organic-compounds-water-capillary-column-gas
