# Supplementary material for: Beyond cyanotoxins: increased Legionella, antibiotic resistance genes in western Lake Erie water and disinfection-byproducts in their finished water
Source: Front Microbiol. 2023 Aug 28;14:1233327. doi: 10.3389/fmicb.2023.1233327 (PMC10493389; doi:10.3389/fmicb.2023.1233327)
Supplement: Supplementary file 1 [file Table_1.DOCX]

**Beyond cyanotoxins: Increased *Legionella,* antibiotic resistance genes in western Lake Erie water and disinfection-byproducts in their finished water**

Jiyoung Lee^1,2,3*^, Seungjun Lee^4^, Chenlin Hu^5^ and Jason W. Marion^6^

^1^College of Public Health, Division of Environmental Health Sciences, The Ohio State University, Columbus, Ohio, USA

^2^Department of Food Science and Technology, The Ohio State University, Columbus, Ohio, USA

^3^Infectious Diseases Institute, The Ohio State University, Columbus, Ohio, USA

^4^Department of Food Science and Nutrition, Pukyong National University, Busan, Republic of Korea

^5^College of Pharmacy, University of Houston, Houston, Texas, USA

^6^Department of Public Health and Clinical Sciences, Eastern Kentucky University, Richmond, Kentucky, USA

*Correspondence: Jiyoung Lee

Address: 1841 Neil Ave, Columbus, OH, USA, 43210, E-mail: [lee.3598@osu.edu](mailto:lee.3598@osu.edu)

| **Table S1.** Summary of water quality parameters measured from two drinking water treatments’ source water and finished water.**Year​** | **Control site​** | | **Bloom site** | |
| --- | --- | --- | --- | --- |
|  | Source​ | Finished​ | Source​ | Finished​ |
| **Temperature (°C)**​ | | | | |
| 2013​ | 14.85±1.15​ | 15.24±1.15​ | 19.92±0.93​ | 19.90±0.72​ |
| 2014​ | 17.59±0.79​ | 17.86±0.75​ | 19.30±0.88​ | 19.56±0.67​ |
| 2013&14​ | 16.07±0.75​ | 16.40±0.74​ | 19.58±0.64​ | 19.71±0.49​ |
| **Turbidity (NTU)**​ | | | | |
| 2013​ | 9.97±2.30​ | 0.12±0.01​ | 15.40±2.68​ | 0.07±0.00​ |
| 2014​ | 10.51±2.72​ | 0.12±0.01​ | 16.76±2.22​ | 0.06±0.00​ |
| 2013&14​ | 10.21±1.76​ | 0.12±0.01​ | 16.13±1.72​ | 0.07±0.00​ |
| **pH**​ | ​ | ​ | ​ | ​ |
| 2013​ | 8.02±0.02​ | 7.30±0.01​ | 8.39±0.09​ | 9.36±0.02​ |
| 2014​ | 7.77±0.03​ | 7.13±0.01​ | 8.25±0.05​ | 9.39±0.02​ |
| 2013&14​ | 7.91±0.02​ | 7.22±0.01​ | 8.32±0.05​ | 9.37±0.02​ |
| **Hardness (mg/L)**​ | | | | |
| 2013​ | 117.14±0.62​ | 116.63±1.87​ | 116.00±1.38​ | 78.75±1.14​ |
| 2014​ | 116.36±0.35​ | 116.29±0.44​ | 118.07±2.42​ | 80.82±1.84​ |
| 2013&14​ | 116.79±0.38​ | 116.48±0.32​ | 117.12±1.57​ | 79.87±1.19​ |
| **Total organic carbon (mg/L)**​ | | | | |
| 2013​ | 2.39±0.05​ | 1.71±0.04​ | 4.02±0.14​ | 1.63±0.04​ |
| 2014​ | 2.49±0.05​ | 1.73±0.04​ | 3.21±0.11​ | 1.42±0.04​ |
| 2013&14​ | 2.43±0.03​ | 1.72±0.03​ | 3.59±0.10​ | 1.52±0.03​ |
| **Nitrate (mg/L)**​ | | | | |
| 2013​ | 0.27±0.04 ​ | 0.17±0.03​ | 0.62±0.15​ | 0.59±0.13​ |
| 2014​ | 0.41±0.05​ | 0.52±0.06​ | 0.66±0.14​ | 0.63±0.13​ |
| 2013&14​ | 0.33±0.03​ | 0.33±0.04​ | 0.64±0.10​ | 0.611±0.10​ |
| **Phosphate (mg/L)**​ | | | | |
| 2013​ | 0.10±0.02​ | 0.73±0.05​ | 0.16±0.02​ | 0.38±0.03​ |
| 2014​ | 0.08±0.03​ | 0.80±0.04​ | 0.13±0.01​ | 0.61±0.08​ |
| 2013&14​ | 0.09±0.02​ | 0.76±0.03​ | 0.14±0.01​ | 0.51±0.05​ |
